# A Comprehensive Three-Dimensional Simulation Method for Skull Base Meningioma Surgery

**DOI:** 10.7759/cureus.106045

**Published:** 2026-03-28

**Authors:** Kohei Ishikawa, Kentaro Watanabe, Nobuyuki Watanabe, Toshihide Tanaka, Yuichi Murayama

**Affiliations:** 1 Department of Neurosurgery, The Jikei University School of Medicine, Tokyo, JPN

**Keywords:** meningioma, neurosurgical education, skull base surgery, surgical simulation, three-dimensional imaging

## Abstract

Preoperative planning for skull base meningioma surgery requires a detailed understanding of complex anatomical relationships and careful strategy development. Although three-dimensional (3D) simulation has been increasingly used for surgical planning, conventional simulations mainly visualize anatomical structures and rarely reproduce the sequential process of tumor removal. We developed a method to perform a near-complete digital 3D simulation of skull base meningioma surgery using a surgical simulation application.

Preoperative imaging data in Digital Imaging and Communications in Medicine (DICOM) format were imported into a simulation platform that automatically generated 3D models of the brain, vasculature, skull, cranial nerves, and tumor through artificial intelligence-based segmentation. Additional reconstruction techniques were applied to create a virtual dura mater. By combining built-in functions for tissue retraction and drill-based removal and labeling the tumor as a removable structure, the simulation reproduced key surgical steps, including craniotomy, tumor detachment from the dural attachment, internal debulking, and dissection from surrounding neurovascular structures.

This method was applied to a 59-year-old woman with a symptomatic anterior clinoidal meningioma compressing the optic nerve and encasing the terminal portion of the internal carotid artery. Preoperative simulation reproduced the planned surgical workflow, including frontotemporal craniotomy, anterior clinoidectomy, tumor detachment, compartmental debulking, and dissection from adjacent vessels and the optic nerve. Intraoperative findings closely corresponded to the simulated procedure, and gross total resection was achieved without neurological complications.

Near-complete digital simulation of skull base meningioma surgery may facilitate visualization of operative strategies and procedural steps in complex cases. This approach has the potential to serve as a useful tool for surgical planning and neurosurgical training.

## Introduction

Resection of skull base meningiomas is one of the most technically demanding procedures in neurosurgery because of complex skull base anatomy, limited surgical corridors, and close relationships with critical neurovascular structures. Although advances in microsurgical techniques and imaging modalities have improved surgical outcomes in recent years, safe and effective tumor removal still requires thorough preoperative anatomical understanding and careful surgical strategy planning.

Three-dimensional (3D) simulation has increasingly played an important role in preoperative planning and neurosurgical education [1-4]. Its advantages include the ability to visualize the operative field from any angle without interference from blood, cerebrospinal fluid, or soft tissues, as well as to understand the anatomical relationships of important structures that may be hidden during actual surgery. Conventional 3D simulations have mainly been used to visualize anatomical relationships and to anticipate the operative field [1,4,5]. However, reproducing the actual process of tumor removal, including detachment from the dural attachment, tumor debulking, and dissection from surrounding structures, has remained challenging. As a result, conventional simulations have been limited in their ability to reproduce surgical procedures in a manner that closely reflects real operative workflows.

In this study, we developed a novel method for near-complete 3D simulation of skull base meningioma surgery using a surgical simulation application (GRID, Kompath Inc., Tokyo, Japan). We report this method with a representative clinical case, demonstrating the feasibility of simulating nearly all surgical steps within a digital 3D environment.

## Case presentation

Preoperative imaging data were exported in Digital Imaging and Communications in Medicine (DICOM) format and imported into a surgical simulation application (GRID, Kompath Inc., Tokyo, Japan). Based on the DICOM data, GRID automatically generated 3D models using artificial intelligence-based segmentation of normal anatomical structures, including the brain, vasculature, and skull.

Imaging studies included non-contrast CT for construction of the skull, brain, and dura mater; MRI with constructive interference in steady state (CISS) sequences for generation of cranial nerves; time-of-flight MRA (TOF-MRA) for construction of intracranial arteries; and contrast-enhanced axial T1-weighted MRI for delineation of the tumor. For the construction of the dura mater, we previously reported a method for creating a virtual tentorium in 3D simulations [6]. This method involves indirect generation of the dura by extracting a region of interest corresponding to a structure whose outer surface geometry matches the tentorium and then applying a subtraction process. The same approach was applied to reconstruct the convexity dura. Because the convexity dura corresponds to the inner surface of the skull, regions of interest, including all intracranial structures, were extracted. This structure was duplicated, slightly reduced in size, and subtracted, leaving a virtual dural layer as the outer shell.

GRID is equipped with built-in functions that allow tissue retraction and bone removal, either by region-based deletion or drill-based removal. By applying these functions and intentionally labeling the tumor as a drillable structure, tumor removal becomes possible, enabling simulation of nearly all procedural steps of meningioma surgery, including skull base surgical techniques. Step 1: Craniotomy was performed using region-based bone removal, and skull base surgical techniques were added as needed. Step 2: Tumor detachment and devascularization were achieved by removal of the tumor tissue at the dural attachment using a small drill, allowing detachment of the tumor from the dura mater. Step 3: Tumor debulking was performed by removing tumor tissue from within the tumor using a larger drill. Step 4: The tumor was dissected from the surrounding brain by applying tissue retraction to separate the tumor from adjacent neural structures.

Using this method, a simulation was performed for an actual case of skull base meningioma, followed by the corresponding surgical procedure. The steps that can be fully simulated and those that cannot be simulated or are only partially reproduced using this method are summarized in Tables 1, 2.

**Table 1:** Surgical workflow that can be reproduced in the digital three-dimensional simulation. The table summarizes surgical steps that can be fully simulated, partially simulated, or not reproduced in the current simulation environment.

Surgical step	Fully simulated	Partially simulated	Not simulated
Craniotomy	✓		
Bone drilling	✓		
Tissue retraction	✓		
Skull base approach (clinoidectomy, petrosectomy)		✓	
				Dura propria elevation			✓
"4D" sequence of meningioma surgery (detachment, devascularization, debulking, dissection)	✓		

**Table 2:** Anatomical structures that cannot be fully reproduced in the digital three-dimensional simulation. The table summarizes anatomical limitations of the current simulation method.

Structure or factor	Fully simulated	Partially simulated	Not simulated
Convexity dura	✓		
Cerebellar tentorium		✓	
Cavernous sinus dura		✓	
Bilayer structure of the dura mater			✓
Relationship between dural folds and tumor			✓
Small vessels and cranial nerves not visible on routine imaging			✓
Tumor consistency			✓
Tumor-brain adhesion			✓

A 59-year-old woman presented with progressive visual impairment. Brain MRI revealed an extra-axial tumor with a maximum diameter of 24 mm located at the left anterior clinoid process, compressing the optic nerve. The terminal portion of the left internal carotid artery was encased by the tumor. Based on the diagnosis of symptomatic anterior clinoidal meningioma, microsurgical tumor resection was planned.

Preoperatively, a 3D simulation was performed using GRID (Video 1). A frontotemporal craniotomy was performed, and the dura mater was retracted to expose the tip of the anterior clinoid process, which was removed extradurally. During intradural procedures, the Sylvian fissure was dissected, and the frontal and temporal lobes were gently retracted to obtain adequate working space. Tumor tissue along the dural attachment was removed using a small drill to achieve detachment. The surface of the tumor was drilled to expose the encased internal carotid artery. The tumor was divided into three compartments surrounded by the internal carotid artery, M1 segment of the middle cerebral artery, and A1 segment of the anterior cerebral artery. Tumor debulking was performed in each compartment, followed by careful dissection from surrounding critical structures, including the internal carotid artery and optic nerve, allowing compartment-by-compartment tumor removal.

**Video 1:** Digital three-dimensional simulation of anterior clinoidal meningioma surgery. The video demonstrates the digital simulation of the surgical workflow in this case. ACP: anterior clinoid process; ICA: internal carotid artery; MCA: middle cerebral artery; ACA: anterior cerebral artery; SV: Sylvian vein; ON: optic nerve; CN III: oculomotor nerve.

**Figure 1:**
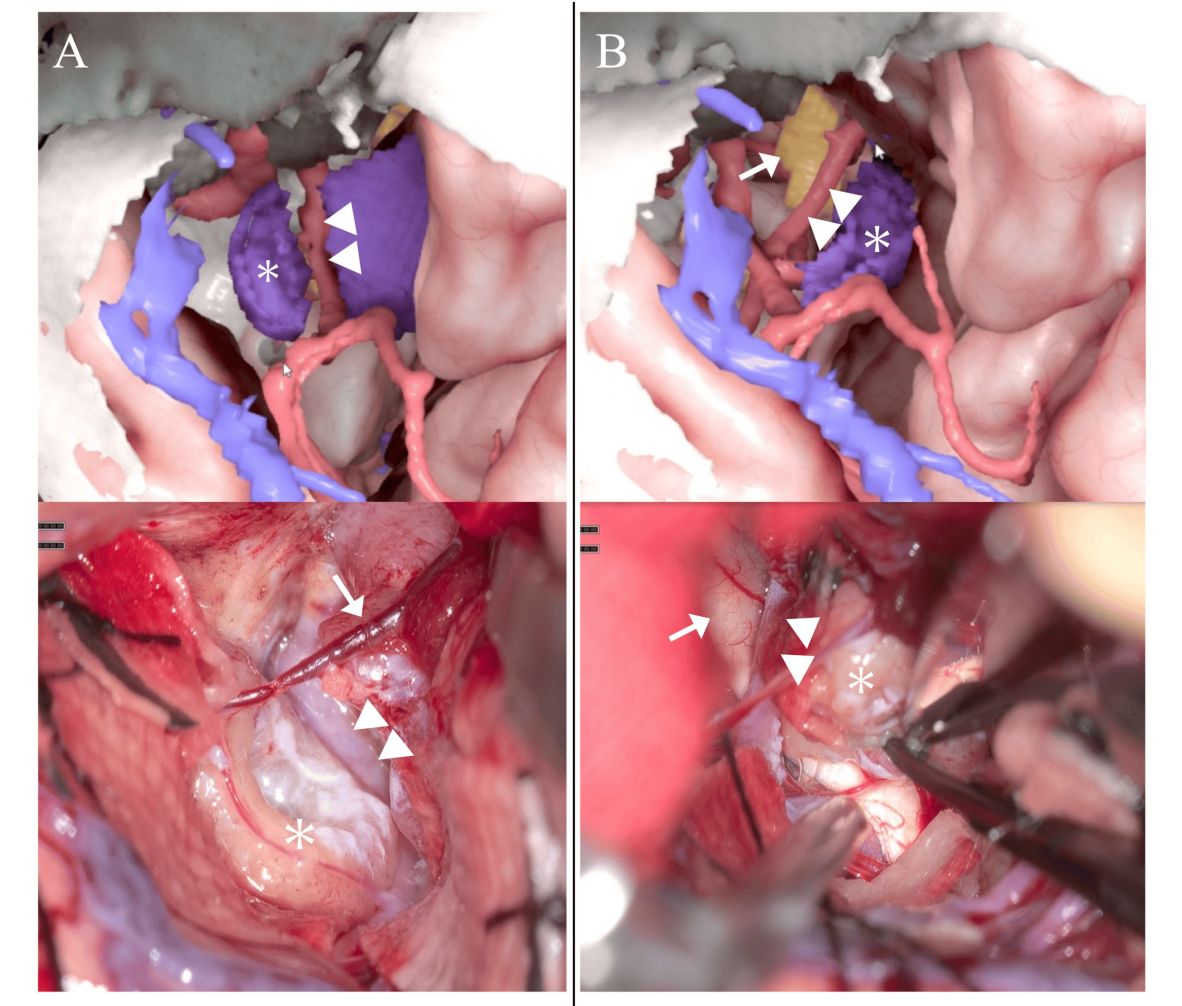
Resection of a left anterior clinoidal meningioma. The upper panels show the digital simulation, and the lower panels show the corresponding intraoperative views. A: The tumor (asterisk) is divided into compartments by major vessels (arrowheads indicate the internal carotid artery), and debulking is performed in the lateral compartment. In the digital simulation, debulking is represented by removing tumor tissue from within the tumor using a drill, creating an eggshell-like cavity. In the simulation, small vessels running over the tumor surface (arrow) are not depicted. B: Dissection is performed. In the digital simulation, this step is represented by retracting the tumor (asterisk) while keeping the surrounding structures fixed. During retraction, the simulation also allows assessment of the relationship between the tumor and surrounding arteries (arrowheads indicate the anterior cerebral artery) and cranial nerves (arrow indicate optic nerve).

In the actual surgical field, small arteries and veins were observed running on the tumor surface; otherwise, intraoperative findings were consistent with the preoperative simulation (Figure 1). After detachment from the dural attachment and devascularization, debulking and dissection were performed for each compartment, achieving gross total resection. Histopathological examination confirmed the diagnosis of meningothelial meningioma. The postoperative course was uneventful, and the patient was discharged without neurological complications.

## Discussion

**Figure 2:**
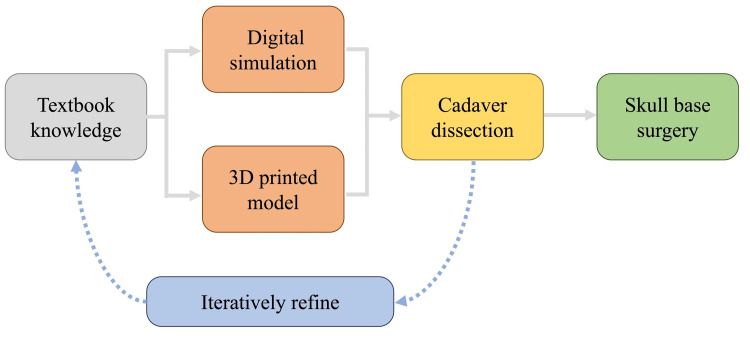
Flowchart for acquiring skull base surgical skills. Digital simulation is proposed as a preparatory step before cadaver dissection. Each step is iteratively refined to acquire anatomical knowledge and technical skills before proceeding to actual surgery. Figure 2 was created by author Kohei Ishikawa using Microsoft PowerPoint (Microsoft Corporation, Redmond, Washington, United States).

In this study, we reported a method for performing a near-complete 3D simulation of the entire surgical workflow for skull base meningioma resection, demonstrated through a representative case of clinoidal meningioma. As surgical techniques and knowledge continue to advance, neurosurgical procedures are increasingly expected to achieve higher levels of safety and efficacy. At the same time, opportunities for young neurosurgeons to gain hands-on experience with complex skull base surgical techniques are likely to decrease, highlighting the need for effective training methods. We have previously reported a technique for creating virtual dura mater, including the tentorium cerebelli, in 3D simulations [6]. When combined with the characteristics of GRID, this approach enables simulation of skull base surgical techniques such as anterior clinoidectomy and petrosectomy by allowing retraction of the dura mater to expose the skull base and subsequent bone removal. Because the virtual dura mater is represented as a single layer, elevation of the dura propria cannot be reproduced. Nevertheless, this simulation allows understanding of the sequence of bone removal, surgical viewing angles, and surrounding anatomy, which are essential components of skull base surgery. Cadaver dissection remains essential for training in skull base surgery; however, opportunities for such training are often limited because of high costs and restricted availability of donated specimens. In this study, we proposed a surgical training method based on digital simulation. Over the past decade, surgical simulation using 3D-printed models has been increasingly reported in the literature, and multi-material models with variable stiffness have enabled hands-on practice of surgical techniques [7,8]. We believe that combining these approaches may contribute to improved anatomical understanding and technical proficiency (Figure 2). Although the actual educational and clinical impact of this approach requires further validation, performing 3D simulation of skull base surgery has the potential to enhance the quality and safety of surgical treatment.

Meningioma resection generally follows a consistent sequence of surgical steps regardless of tumor location, commonly described as the “4D” concept: detachment, devascularization, debulking, and dissection [9,10]. GRID provides built-in functions that allow tissue retraction, as well as bone removal, performed either by region-based deletion or drill-based removal. By intentionally labeling the segmented tumor as a drillable structure, we applied these functions to reproduce the 4D surgical concept in the simulation. Detachment and devascularization were simulated by removing tumor tissue at the dural attachment, debulking by drilling tumor tissue from within the tumor, and dissection by retracting the tumor away from the adjacent brain. Because the simulation can be repeated multiple times, it allows visual sharing of surgical strategies and resection sequences with expert surgeons. This feature is particularly useful and educational for complex cases such as clinoidal meningiomas with encasement of the internal carotid artery, in which compartment-based surgery is required.

Despite these capabilities, several limitations of this simulation method should be acknowledged. First, regarding the surgical workflow, in this method, the tumor is intentionally labeled as a drillable structure to enable simulated removal. As a result, if the tumor is not completely separated from the surrounding bone, retraction applied to the tumor may simultaneously affect adjacent bony structures. Depending on the tumor location, complete simulation of the surgical procedure may therefore be difficult. Future software updates that allow direct removal of tumor tissue may help overcome this limitation. Second, regarding anatomical limitations, accurate construction of the dura mater in the cavernous sinus region remains challenging with the current technique. Further advances in artificial intelligence-based segmentation are required to address this issue. Third, small vascular structures that are not visualized on routine imaging studies cannot be constructed in the simulation. In the present case, small perforating arteries and thin arteries and veins running on the tumor surface were not visualized. This limitation may be mitigated in the future by improving imaging resolution through the use of cerebral angiography or high-field magnetic resonance imaging, such as 7-T MRI. Finally, the simulation cannot reproduce tumor consistency or the degree of adhesion between the tumor and surrounding brain tissue. Accumulation of data comparing preoperative imaging findings with intraoperative observations may enable future AI-based prediction of these properties. Despite these limitations, performing skull base meningioma surgery not only in actual clinical cases but also through digital 3D simulation enables a clear understanding of skull base surgical techniques, procedural steps of meningioma removal, and overall surgical strategy.

## Conclusions

We reported a method for performing a near-complete digital 3D simulation of skull base surgery. This approach allows visual understanding of surgical strategies for complex skull base tumors and may support preoperative planning and surgical education. By reproducing key procedural steps in a digital environment, the simulation provides an intuitive understanding of anatomical relationships and operative workflow. Further studies are warranted to evaluate its educational and clinical impact in neurosurgical training and practice.
